# Protection induced by a glycoprotein E-deleted bovine herpesvirus type 1 marker strain used either as an inactivated or live attenuated vaccine in cattle

**DOI:** 10.1186/1746-6148-10-8

**Published:** 2014-01-08

**Authors:** Sonia Alejandra Romera, Mariana Puntel, Valeria Quattrocchi, Paula Del Médico Zajac, Patricia Zamorano, Javier Blanco Viera, Consuelo Carrillo, Shafiqul Chowdhury, Manuel V Borca, Ana M Sadir

**Affiliations:** 1 Instituto de Virología Instituto de Virología, Centro de Investigaciones en Ciencias Veterinarias (CICV), Instituto Nacional de Tecnología Agropecuaria (INTA), Castelar, CC77, 1708 Morón, Argentina; 2 Fundacion Instituto Leloir-IIBBA, CONICET, Av. Patricias Argentinas 435, 1405 CABA, Argentina; 3 Instituto de Patobiología-CICVyA, INTA, Castelar CC77, 1708 Morón, Argentina; 4 Plum Island Animal Disease Center, APHIS, USDA, Greenport, NY 11944 USA; 5 Department of Pathobiological Sciences, School of Veterinary Medicine, Louisiana State University, Baton Rouge, LA 70803 USA; 6 Plum Island Animal Disease Center, ARS, USDA, Greenport, NY 11944 USA; 7 Universidad del Salvador, Buenos Aires, Argentina; 8 CONICET, Buenos Aires, Argentina

**Keywords:** BoHV-1ΔgEβgal, BoHV-1 marker vaccine, Inactivated/live attenuated vaccine, Bovines

## Abstract

**Background:**

Bovine herpesvirus type 1 (BoHV-1) is the causative agent of respiratory and genital tract infections; causing a high economic loss in all continents. Use of marker vaccines in IBR eradication programs is widely accepted since it allows for protection of the animals against the disease while adding the possibility of differentiating vaccinated from infected animals.

The aim of the present study was the development and evaluation of safety and efficacy of a glycoprotein E-deleted (gE-) BoHV-1 marker vaccine strain (BoHV-1ΔgEβgal) generated by homologous recombination, replacing the viral gE gene with the β-galactosidase (βgal) gene.

**Results:**

*In vitro* growth kinetics of the BoHV-1ΔgEβgal virus was similar to BoHV-1 LA. The immune response triggered by the new recombinant strain in cattle was characterized both as live attenuated vaccine (LAV) and as an inactivated vaccine. BoHV-1ΔgEβgal was highly immunogenic in both formulations, inducing specific humoral and cellular immune responses. Antibody titers found in animals vaccinated with the inactivated vaccine based on BoHV-1ΔgEβgal was similar to the titers found for the control vaccine (BoHV-1 LA). In the same way, titers of inactivated vaccine groups were significantly higher than any of the LAV immunized groups, independently of the inoculation route (p < 0.001). Levels of IFN-γ were significantly higher (p < 0.001) in those animals that received the LAV compared to those that received the inactivated vaccine. BoHV-1ΔgEβgal exhibited an evident attenuation when administered as a LAV; no virus was detected in nasal secretions of vaccinated or sentinel animals during the post-vaccination period. BoHV-1ΔgEβgal, when used in either formulation, elicited an efficient immune response that protected animals against challenge with virulent wild-type BoHV-1. Also, the deletion of the gE gene served as an immunological marker to differentiate vaccinated animals from infected animals. All animals vaccinated with the BoHV-1ΔgE βgal strain were protected against disease after challenge and shed significantly less virus than control calves, regardless of the route and formulation they were inoculated.

**Conclusions:**

Based on its attenuation, immunogenicity and protective effect after challenge, BoHV-1ΔgEβgal virus is an efficient and safe vaccine candidate when used either as inactivated or as live attenuated forms.

## Background

Bovine herpesvirus-1 (BoHV-1) is an important pathogen of cattle and responsible for a wide variety of clinical diseases, including conjunctivitis and upper respiratory tract infection known as infectious bovine rhinotracheitis (IBR), reproductive tract lesions, abortion in pregnant cows, and systemic infection in the newborn [1-3]. In addition, BoHV-1 has been recognized as an important component of the bovine respiratory disease (BRD) complex [1,4]. The BoHV-1 infection in cattle is responsible for considerable economic losses due to decreased milk production, weight loss and abortion.

The virus genome consists of a linear double-stranded DNA molecule of about 135.3 kb that encodes for approximately 70 proteins [5]. Twelve of them (gB, gC, gD, gE, gG, gI, gH, gK, gL, gM, UL49.5 and Us9) are envelope proteins, of which the first ten are glycosylated [5]. Envelope glycoproteins gE and gI form complexes and gE is involved in viral intercellular spread (cell-to-cell spread). BoHV-1 gE open reading frame (ORF) is predicted to contain 575 amino acid (aa) residues with a 28 aa cleavable signal sequence. The structure of glicoprotein E (gE) corresponds to a type I transmembrane glycoproteins. The glycoprotein contains three distinct domains: a 387 aa long hydrophilic extracellular domain (ecto-domain), a 33 aa long hydrophobic transmembrane domain and a 125 aa long highly charged cytoplasmic domain/tegument domain. The mature gE is phosphorylated and glycosylated with an apparent molecular mass of about 92 kD.

Attenuated live viruses or inactivated virions are used widely as vaccines to control the disease. Thus, BoHV-1-specific antibodies can be found in bovines on all continents; its prevalence varies greatly depending on herd size and management. Classical vaccines complicate serological diagnosis and determination of the true prevalence of infection. Marker and conventional vaccines can prevent disease but not latent infection. Due to the current restrictions in international trade for products derived from seropositive animals a number of European countries have eradicated BoHV-1 with very high costs involved. In countries with a high prevalence of infection, including the United States, the control of IBR is associated with the immunization of cattle with marker vaccines. In this context, the ability to differentiate infected from vaccinated animals (DIVA) has become critical issue. Gene-deleted marker vaccines offer the advantage of deletion of specific viral genes that are non-essential for viral replication [6,7] while inducing a substantial protein-specific immune response. Currently, vaccines used in BoHV-1 control programs utilize highly attenuated BoHV-1 strains marked by a deletion of the gE gene [8]. Glycoprotein E is considered a virulence factor of all known members of the subfamily *Alphaherpesvirinae*[9-11].

Live and killed gE-deleted marker vaccines are now widely used in Europe, in combination with gE-based diagnostic tests to monitor cattle. The gE-deleted mutants generated thus far include derivations of herpes simplex virus (HSV), pseudorabies virus, equine herpesvirus 1 and BoHV-1; these were shown to be attenuated in mice, swine, foals and calves, respectively [8,9,12,13]. Notably, expression of gE and gI is required for full pathogenic potential in animals but is not required for growth in tissue culture [14], thus allowing for it replication in cell culture. In infected animals, gE is required for anterograde neuronal spread and neurovirulence. Accordingly, an interesting safety feature that comes from using LAV based on gE-deleted viruses is that the virus cannot spread in an anterograde direction from sensory neurons in trigeminal ganglia to respiratory mucosa. Furthermore, gE induces specific antibodies both in the context of inactivated vaccines or as infectious virus, thus facilitating serological differentiation of animals immunized with a gE-deleted marker vaccine from infected cattle [7]. Safety and efficacy of both the live and the inactivated gE-deleted vaccines that are available have been tested thoroughly [15-22].

In this report, we present data regarding the development of a gE-deleted and βgal expressing BoHV-1 (BoHV-1ΔgEβgal) strain and its use as either an inactivated immunogen or as an LAV in cattle. Our results demonstrate that BoHV-1ΔgEβgal virus is an efficient vaccine candidate when used either as inactivated or as live attenuated form.

## Methods

### Virus and cells

BoHV-1 Los Angeles (LA) (American Type Culture Collection, VR-188) and BoHV-1ΔgEβgal strains were propagated in Madin Darby Bovine Kidney (MDBK) cells grown in Eagle’s Minimal Essential Medium (MEM), supplemented with 10% fetal bovine serum (FBS).

### Construction of the recombinant plasmid

Virus DNA was extracted from cell-free supernatant virus preparations following a standard phenol/chloroform procedure using sodium dodecyl sulfate (SDS) and proteinase K lysis, phenol/ether extraction, and ethanol precipitation [23]. Discrete flanking regions corresponding to gE gene were amplified by PCR from this material. Primer sequences were based on the published BoHV-1 glycoprotein E (gE) gene sequences [GenBank Accession Number NC 001847]. Primers were designed to add restriction sites in order to facilitate further cloning. The left flanking region of gE (L fragment) was obtained using primers gE1 (5′-GCGAGCAGCGGGAGCGGGGCC-3′),; and gE2(5′-GGGGCGGATCCGTGGGTTGCA-3′), which introduced a Sal I and a Bam HI sites, respectively. The right flanking region of gE (R fragment) was amplify using primers gE3(5′- AGCTTGGATCCCGGCCGCACC- 3′) and gE4 (5′- CCTCAGAATTCGGGGTCTCGG- 3′), which introduced a Bam Hi and an EcoR I sites to amplified fragment, respectively.

DNA fragments corresponding to the left (L) and right (R) gE flanking regions obtained by PCR were digested with the appropriate enzymes and cloned sequentially into the pUC19 vector (Clontech). First, the L product (734 bp long, corresponding to position 120956 to 121714 of the BoHV-1 genome) was cloned between the SalI and BamHI sites (recombinant plasmid pUCL), and then the R product (632 bp long, corresponding to position 123375 to 124008 of the BoHV-1 genome) was cloned between the BamHI and EcoRI sites (recombinant plasmid pUCLR), using a BamHI site that linked both L and R fragments. The blunt 4.5 kb fragment, which contains the bacterial β-gal gene under the control of the human cytomegalovirus immediate early promoter (HCMV-IE), was inserted into the blunted BamHI site of pUCLR. The resulting gE-deletion/β-gal insertion plasmid (pUCLRβ-gal) carried a deletion of 1,640 bp of BoHV-1 DNA, and the insertion of the β-gal gene controlled by the human cytomegalovirus immediate early promoter. In pUCLRβ-gal, the β-gal gene is flanked by virus-specific sequences required for recombination with the viral DNA: a 734 bp upstream sequence (L) containing part of the gE promoter and the first 23 bp of the coding sequence, a 632 bp downstream sequence (R) containing the last 67 bp at the 3′ extreme, and the complete ORF of the US9 gene (474 bp). Identity of both fragments corresponding to L and R were confirmed by sequencing (Fmol, Promega) (data not shown).

### Generation of recombinant BoHV-1 gE-deleted βgal (BoHV-1ΔgEβgal) virus

To generate the BoHV-1 gE-deleted recombinant virus, MDBK cells were co-transfected with a ScaI linearized pUCLRβ-gal plasmid DNA together with a full-length DNA derived from BoHV-1 LA strain. Briefly, a mixture of 2 μg of parental BoHV-1 LA DNA, 0.2 μg of ScaI linearized pUCLRβgal vector and 16 μl of Lipofectamine (Gibco BRL, Life Technologies) was added onto a 60% confluent MDBK cell monolayergrown in a 6-well plate, and then incubated for 8 hours at 37°C in a CO_2_-controlled atmosphere. A media change was then performed using MEM containing 5% FBS and the plates were incubated for 16 hours in the same conditions described above. Each transfected cell monolayer was trypsinized and transferred to a 25 cm^2^ flask containing 5% FBS MEM and incubated until cytopathic effects (CPE) were observed. To determine the dilution of the inoculum for the Bluo-gal screening assay, the co-transfection products were titrated by plaque forming units (PFU) and frozen until the screening assay was performed.

### Purification of the recombinant virus

Co-transfection products were examined for their β-gal expression by histochemical staining of infected cells. The recombinant viruses able to form blue plaques in the presence of the β-galactosidase substrate (halogenated indolyl-β-D-galactoside) were plaque purified five times under an agar overlay [24]. Recombinant viruses expressing β-gal activity were selected by the appearance of blue plaques.

### Molecular characterization of the recombinant virus

The recombinant virus was further characterized by PCR (gE-specific primers: gE7: 5′-CGCCCGTCTTTCTCCCAG-3′ and gE8: 5′-GCGGGACGAGGAGAGGGA-3′) and by Southern blot analysis targeting a part of the gE ORF. Lack of gE protein expression in BoHV-1ΔgEβgal infected cell lysates done by Western blot.

### Southern blot hybridization

Genomic DNA extracted from purified wild-type and recombinant BoHV-1 viruses were digested with HindIII and the corresponding fragments were separated by electrophoresis in a 0.6% agarose TBE gel, then transferred to a nitrocellulose membrane (Zprobe, Bio-Rad). [25]. The membrane bearing the immobilized DNA fragments was blocked with 2x SSC solution and fixed by heating at 80°C for 30 minutes. Hybridization was performed separately with three specific probes (L, R, y gE) previously obtained by PCR and labeled with 32S (Prime-a-Gene Labeling System, Promega).

### gE-specific immunoblot analyses

Polyacrylamide gel electrophoresis in the presence of SDS (SDS-PAGE) and additional immunoblot analyses were performed using mock and virus-infected cells. Briefly, either mock (non-infected MDBK cells) and virus-infected cells were concentrated by ultracentrifugation at 12,000 g for 1 h at 47°C, resuspended in sample buffer (50 mM Tris–HCl, pH 7.5, 1 mM PMSF, 8 M urea, 1% SDS, 2 mM DTT and 2% β-Mercaptoethanol), boiled for 10 minutes, separated by gel electrophoresis in 12.5% polyacrylamide gels and blotted on to Immobilon P membranes (Millipore). Membranes were blocked overnight in phosphate buffered saline and 0.05% Tween 20 (PBST) containing 5% skim milk (all subsequent steps were performed using this buffer). Then, they were incubated for 2 hours at 37°C with the corresponding monoclonal antibodies: anti-gE, MAb3 (kindly provided by Dr. J.T. Van Oirschot from the Institute of Animal Science and Health, Lelystad, Netherlands), or anti-gD (kindly provided by Dr. L.A. Babiuk from the Vaccine and Infectious Disease Organization, University of Saskatchewan, Saskatoon, Saskatchewan, Canada). After three washes with PBST, membranes were incubated with peroxidase-labeled anti-mouse IgG rabbit antiserum (KLP Inc) for 1 hour at 37°C. After washing three times, antibody binding was visualized by chemiluminescence (Renaissance kit, NEN Life Science) after exposure to an X-ray film (CurixOrto ST-G2, AGFA).

### Virus growth kinetics

The growth kinetics of the BoHV-1ΔgEβgal strain in MDBK cells was compared to that of the parental BoHV-1 LA strain. A series of replicate cultures of MDBK cells were infected separately with a MOI of 0.1 per cell of recombinant BoHV-1ΔgEβgal or the parental BoHV-1 LA strain. Infected cultures were harvested at successive post-infection intervals and frozen in aliquots until titration. Virus titration was performed by the end-point dilution method of Reed & Muench, in three independent repetitions.

### Inactivated vaccine formulation

BoHV-1 LA or BoHV-1ΔgEβgal were propagated according to the conditions described earlier [26]. Preparations were inactivated by treatment with 1% (v/v) 0.1 M binary bromoethylenimine (BEI) for 25 h at 37°C. One volume of inactivated virus suspension was mixed with one volume of INTA mineral oil adjuvant (formulated with Arlacel C, Markol 52 and Tween 80), to produce a water-in mineral oil emulsion according to Smitssart *et al.*[27].

### Vaccination experiments

The animal experiments reported in this manuscript have been performed following internationally recognized guidelines with the approval of the Institutional Committee for Care and Use of Experimental Animals, CICUAE-CICVyA. INTA, Argentina.

Vaccination experiments were performed using 12 to 18 month-old Holando and Angus × Hereford calves with undetectable BoHV-1 serum antibodies (evaluated by ELISA and virus neutralization tests).

All challenge experiments using virulent BoHV-1 LA, and inoculations using attenuated BoHV-1ΔgEβgal (dose: 10^8.25^ TCID_50_/mL) were performed in a type II biosafety animal facility.

Viral challenge was performed using 2 ml virulent BoHV-1 LA (10^7.5^ TCID_50_/ml) into each nostril, by intranasal inoculation (IN) with an ultrasonic nebulizer (ELECTROLAB AP-300).

#### Evaluation of BoHV-1ΔgEβgal as either inactivated and attenuated vaccines

Table 1 shows the experimental design describing number of animals, vaccination date and route, date of challenge, date of BoHV-1 specific antibodies determination, BoHV-1 excretion in nasal fluids, date of lymphoproliferative response.

**Table 1:** Experimental design

**Experiment**	**Vaccine**	**# animals**	**Vaccination time (dpv) [route, vol.]**	**Challenge (dpv)**	**BoHV-1 antibodies (dpv)**	**BoHV-1 sheeding (dpv/dpc)**	**LPT (dpv/dpc)**
inactivated BoHV-1ΔgEβgal vaccine	BoHV-1ΔgEβgal	5	0, 21 [SC, 3 ml]	186	0, 14, 21, 30, 67, 150 and 186	0, 3, 5, 7, 8, 10, 12, 14, 17 and 22 dpc	7,72 dpv
	BoHV-1LA	5	0, 21 [SC, 3 ml]	186			
	mock	6		186			
	
attenuated BoHV-1ΔgEβgal vaccine	BoHV-1ΔgEβgal	5	Group 1: 0 [IN, 4 ml]	42	0, 7, 19, 34 and 42	1 to 42dpv	7,30 dpc
		5	Group 2: 0 [IM, 4 ml]	42		and	
	Sentinel	5	Group 4			3, 5, 7, 8, 10, 12, 14, 17 and 22 dpc	
	Mock	5	Group 5	42			
attenuated BoHV-1ΔgEβgal vaccine SAFETY	BoHV-1ΔgEβgal	5	Group 3: 0 [IV, 4 ml]		along 370		

SC: subcutaneous, dpv: days post-vaccination; dpc: days post-challenge.

During the next 42 days post- vaccination with attenuated BoHV-1ΔgEβgal, viral excretion was controlled, as well as clinical signs such as loss of appetite, lesions of nasal, ocular and oral mucosae and discharge from the nose or eyes and rectal temperature. General clinical status was evaluated and recorded using a rating system (ranging from 0 = asymptomatic to 3 = severe symptoms). Nasal swabs were scored as follows: 0 = absent, 1 = slightly serous, 2 = severely serous, 3 = seromucous, 4 = mucopurulent.

### Safety assessment for BoHV-1ΔgEβgal as a life attenuated vaccine

The safety test was performed in pregnant cows. Five pregnant cows (being in the third to sixth month of pregnancy) were IV infected with attenuated BoHV-1ΔgEβgal virus (4 ml of the viral suspension with a titer of 10^8.25^ TCID_50_/ml). Animals were clinically observed until they gave birth and serologically evaluated until 370 dpv. Likewise, newborn calves were also evaluated in regards to their clinical and immune conditions, as well as the virus shedding.

### Detection of virus neutralizing antibodies

Virus neutralization (VN) was performed using primary fetal bovine testis (FBT) cell cultures in 96-well microtiter plates using the constant virus-variable serum method [26,27]. Each dilution was tested in four wells containing FBT cell monolayers an adsorbed for 1 hour, the unabsorbed virus was removed and MEM with 5% FBS, was added. Plates were then incubated for 3 days at 37°C and the number of wells and serum dilution showing cytopathic effect was scored. Log10 of the reciprocal value of the highest serum dilution in which CPE was prevented was considered the virus neutralization titer.

### Detection of anti-BoHV-1 and anti-gEantibodies in serum

Total antibodies against BoHV-1 in bovine serum were measured by an indirect ELISA as described elsewhere [28]. Titers for bovine sera were expressed as Log10 of the reciprocal of the highest serum dilution which gives readings of absorbance greater or equal to 40% of the positive control measuring at an OD of 405 nm. Detection of gE-specific antibodies was performed using a commercial ELISA for BoHV-1 that allows for the detection of gE-specific antibodies (HerdChek Anti-IBR gE/IDEXX Laboratories), according to the manufacturer’s directions.

### Detection of IgG1/IgA antibodies in nasal swabs

The mucosal immune response was determined using a specific ELISA for the detection of IgG1 and IgA. Briefly, nasal swabs were collected and frozen at -80°C. The antigen was prepared as described before (Romera *et al.*, [26]).

### BoHV-1-specific lymphoproliferation assay

Whole blood samples were collected by venipuncture in syringes containing heparin. Lymphocyte-enriched cells were isolated from the buffy coat as described elsewhere [26] by centrifugation on Lymphoprep (Nycomed Pharma A.S.). Cells were washed twice in RPMI + 5% FBS. UV light-inactivated suspensions of virus-infected MDBK cells (5 μg/ml) and control MDBK cells were used as antigens. The corresponding inactivated antigen was added to a 100 μl volume of medium containing 2 × 10^5^ lymphocytes, performed in triplicate in a 96-well plate format. One microgram of the mitogen Concavalin A (Sigma, St Louis, USA) was added to the positive control wells. The lymphocyte cultures were incubated for 4 days at 37°C in a humidified atmosphere containing 5% CO_2_. Twenty hours before harvesting, 4 μCi of [3H] tymidine was added to each well. Cell proliferation was measured as [3H] tymidine uptake in a scintillation counter (LKB, Wallac, 1219 Rackbeta) and results were obtained as counts per minute (CPM). The values are the mean of the triplicate values and are expressed as stimulation index (SI), where SI = mean CPM of antigen-stimulated lymphocytes/mean CPM of MDBK-stimulated lymphocytes). A threshold value of SI = 3 was established.

### Interferon Gamma assay

The capacity of mononuclear cells to secrete interferon γ (IFNγ) in response to a specific *in vitro* stimulation with inactivated BoHV-1 viral antigen was evaluated by a indirect sandwich ELISA. Briefly, 1.5 × 10^6^ mononuclear cells were diluted in 100 μl of RPMI 10% FBS per well, in sterile microplates of 96 “U” bottom wells. The supernatant was added on Immulon II microplates, previously sensitized ON with anti bovine IFNγ monoclonal antibody (mAbs), and blocked with PBST 0.1% BSA. A reference curve was performed using IFNγ standard at known concentrations. Detection of the captured IFNγ was done using a rabbit anti-bovine IFNγ serum, diluted in 0.1% PBST-BSA. In order to improve the sensibility of the assay, plates were later incubated with biotin-labeled rat anti-rabbit IgG and developed using disodium p-nitrophenyl phosphate (PPN) as substrate. Optical densities (OD) at A_405_ were measured 25 minutes after the addition of the substrate.

### Statistical analysis

Comparison of the vaccine profiles obtained throughout the experimental period was performed using an analysis of variance (ANOVA) for repeated measures with the Greenhouse and Geisser correction of the significance levels (fixed at 5%). The post-ANOVA comparisons were performed using the Bonferroni test with the same level of significance. All the statistical calculations were performed using the SAS program (release 6.04), following the G.M.L. procedure [29].

## Results

### Construction of a gE deletion vector

**Figure 1:**
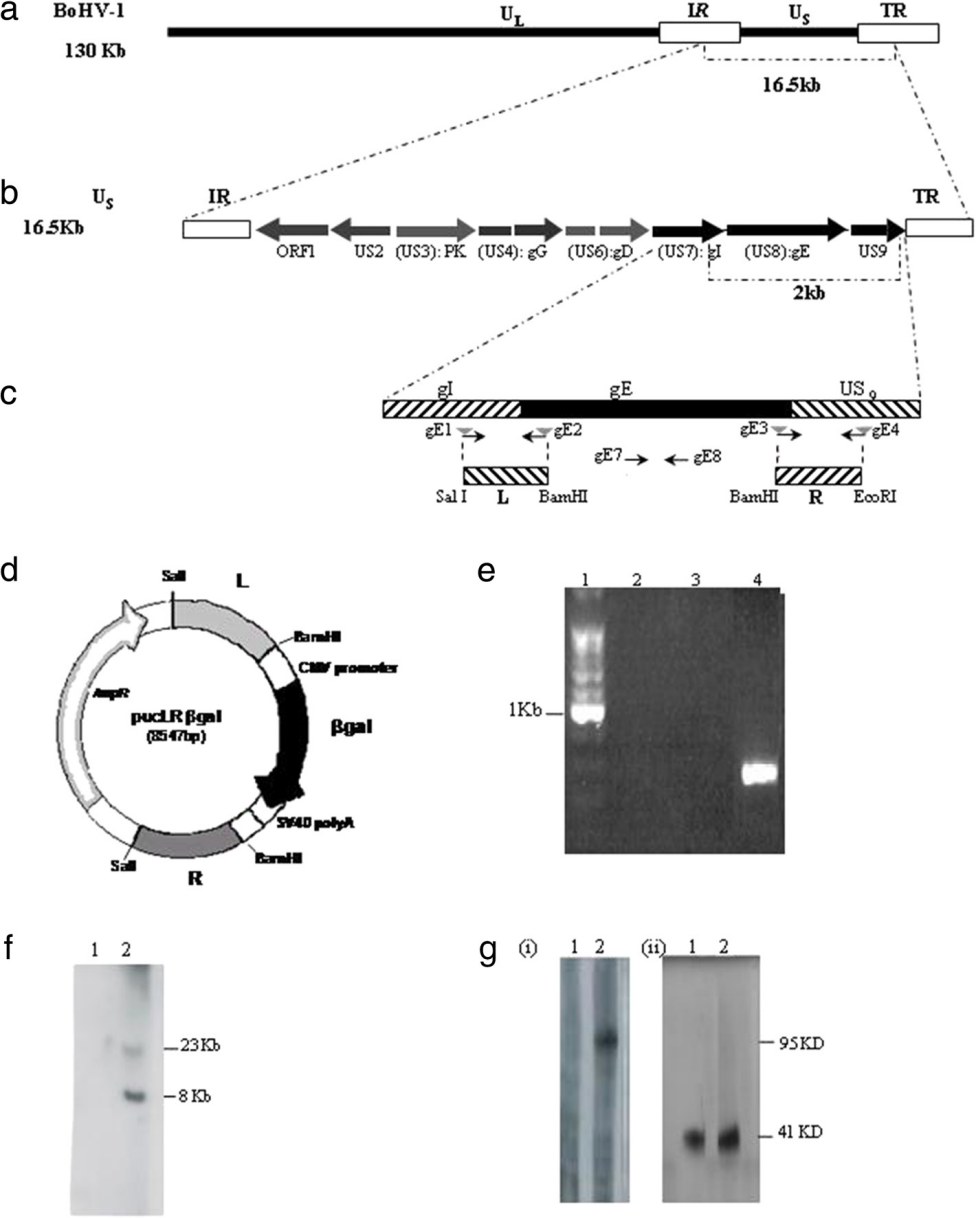
**Molecular structure of BoHV-1ΔgEβgal virus. (a)** Target region for the gE deletion in BoHV-1 genome. **(b)** gE ORF (US8) is flanked by gI (US7) and US9 genes. **(c)** Primers gE1 and gE2 were designed to amplify the left (L) fragment, and primers gE3 and gE4 were designed to amplify the right (R) fragment. The sequences of primers gE2, gE3 and gE4 were designed in order to create the depicted restriction sites to facilitate the cloning strategy. **(d)** pUCLRβgal recombination vector diagram restriction sites used for cloning and relative position of the elements decribed are shown **(e)** Confirmation gE-specific deletion by PCR. Lanes 2–4 primers specific to the gE sequence (gE7 and gE8) were used. Different templates were used for each lane shown: Lane 2 negative control (ultrapure sterile water); lane 3 BoHV-1ΔgEβgal DNA; lane 4 parental BoHV-1 LA DNA; lanes 1 molecular weight marker (100pb, Promega). **(f)** Molecular characterization of the recombinant BoHV-1ΔgEβgal strain. Southern blot analysis for gE gene. Viral DNA from BoHV-1ΔgEβgal (lane 1) and from parental BoHV-1 LA strain (lane 2) were digested with HindIII restriction enzyme, separated by 0.6% agarose gel electrophoresis, blotted to a membrane and probed with the gE probe. **(g)** Western blot for gE protein. Concentrated virus (BoHV-1ΔgEβgal, lane 1; and parental BoHV-1 LA, lane 2) was separated by electrophoresis in a 12% SDS-PAGE, blotted to a membrane and incubated with specific serum. Panel (i) gE-specific monoclonal antibody, panel (ii) gD-specific monoclonal antibody.

The gE gene of BoHV-1 is located between BoHV-1 genome nucleotide positions 121714 and 123440, within the US region (Figure 1a), similarly to other alphaherpesviruses. The BoHV-1 gE gene is flanked upstream by the gI (US7) gene and downstream by the BoHV-1 homologue of the herpes simplex virus (HSV-1) US9 gene [30,31] (Figure 1b). Based on the position of the gE ORF, a gE deletion recombinant vector, pΔgEβgal, was constructed and composed of two DNA fragments flanking the gE coding region (pUCLRβgal), created by PCR amplification of viral DNA from the BoHV-1 LA parental strain (Figure 1d). The upstream flanking fragment is a SalI/BamHI 734 bp region (L) covering from position 120956 to 121714 of the BoHV-1 genome while the downstream fragment is a BamHI/EcoRI 632 bp region (R) covering from position 123375 to 124008 of the BoHV-1 genome (Figure 1c). The βgal marker gene was inserted into the BamHI site of the construct.

### Development of a BoHV-1 gE-deleted virus mutant (BoHV-1ΔgEβgal)

To generate the gE deletion mutant virus, the recombinant vector pUCLRβgal was co-transfected with BoHV-1 LA DNA into MDBK cells. The first screening was based on βgal expression. Selected viruses (blue plaque +) were further evaluated by PCR amplification using specific primers for gE regions (Figure 1e). The intended gE gene deletion and insertion of the βgal gene at the gE locus was further confirmed by Southern blot analysis with a gE-specific probe (Figure 1f). The absence of the 1639 bp gE fragment and the presence of a recombinant βgal fragment with the anticipated size of 4.5 kb demonstrated that the intended recombination had taken place in a site-specific manner. Western blot analysis using a BoHV-1 gE-specific MAb3 confirmed the deletion of the gE protein in the BoHV-1ΔgEβgal virus while a 92- to 95-kd BoHV-1 gE product was detected in BoHV-1 cell extracts (Figure 1g). The L and R fragments of BoHV-1ΔgEβgal were sequenced. No difference with wt virus were found.

**Figure 2:**
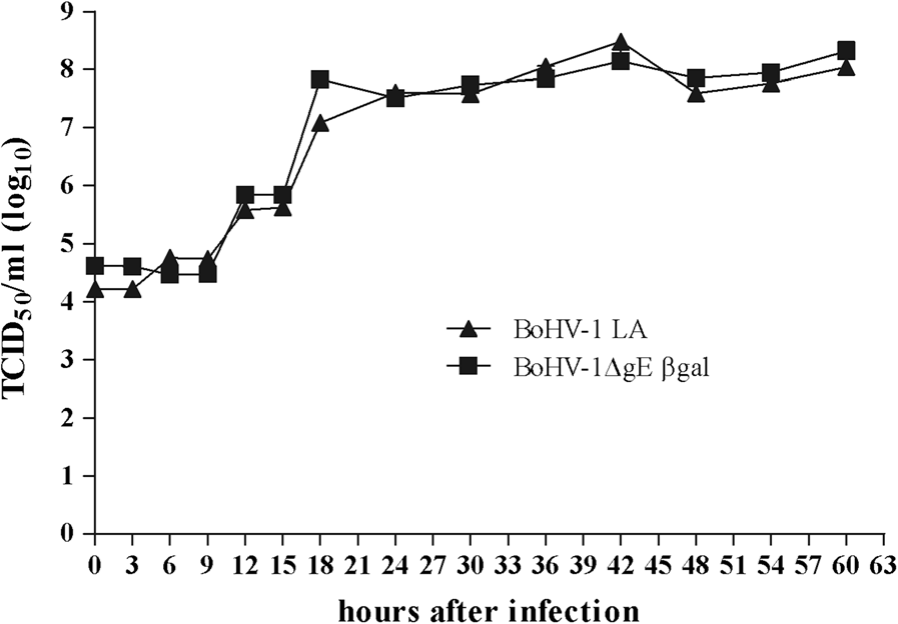
**Growth kinetics of the BoHV-1ΔgEβgal virus.** MDBK cells were infected at a MOI of 0.1 with either BoHV-1ΔgEβgal or BoHV-1 LA virus strains. Viral replication was checked every 3 hours during a period of 60 hours pi. Virus titrations were performed in duplicate for each time point on MDBK cells. Titers are expressed as TCID_50_/ml. Each data point represents the mean of the titers obtained.

Growth properties of the BoHV-1gE deletion mutant virus, BoHV-1ΔgEβgal, were compared with the BoHV-1 LA strain in a multistep growth curve performed using MDBK cell cultures infected at a MOI of 0.1. Virus yields were determined by titration performed using MDBK cells. No significant differences were detected between the titers achieved with wt BoHV-1 strain and the gE-deleted strain (Figure 2).

### Assessment of BoHV-1ΔgEβgal virus as an inactivated vaccine

Animals (n = 5/group) were subcutaneously vaccinated at days 0 and 21 with either a vaccine formulated with BoHV-1ΔgEβgal or wild-type BoHV-1 LA strain. At 186 dpv, the two vaccinated groups and a mock-vaccinated group (n = 6) were IN challenged with 10^7.5^ TCID_50_/ml virulent BoHV-1 LA strain by aerosol exposure.

**Figure 3:**
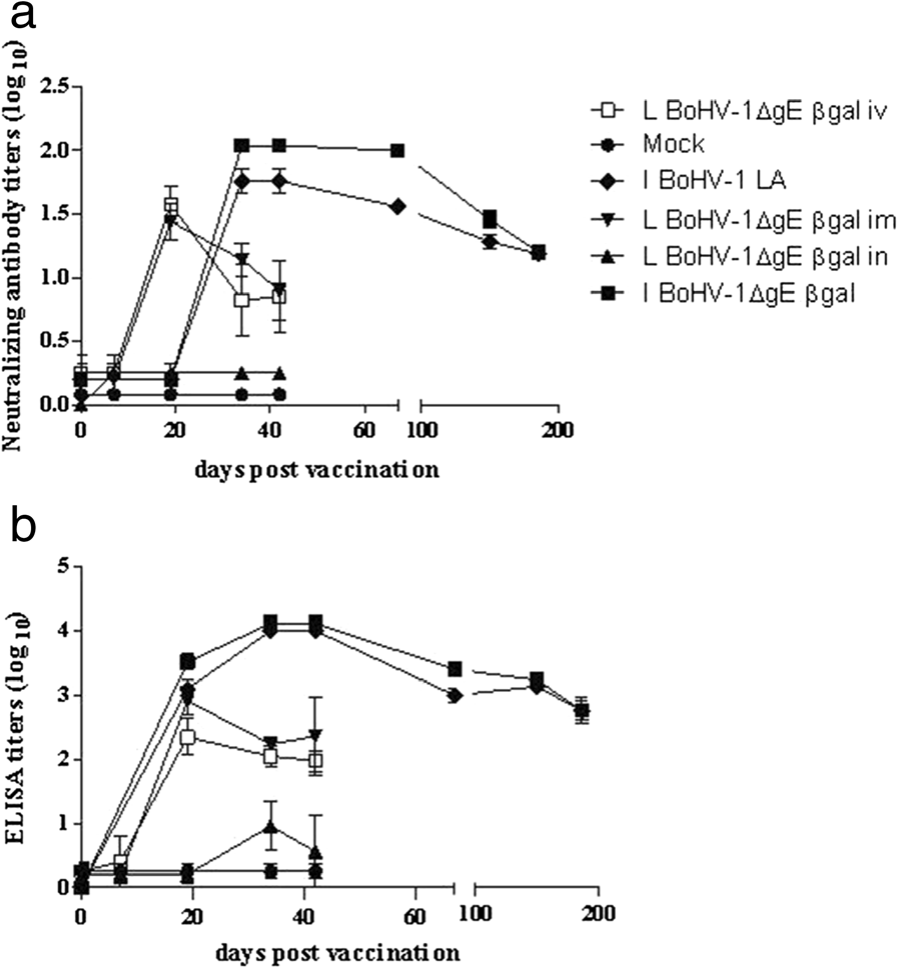
**Antibody response to BoHV-1 observed in vaccinated cattle.** BoHV-1-specific antibody response detected by ELISA **(b)** or virus neutralization assay **(a)** in animals vaccinated either with inactivated (I) or live attenuated vaccine (L) formulated with BoHV-1ΔgEβgal or BoHV-1 LA viruses. Live attenuated vaccines were administered by intranasal (in), intramuscular (im) or intravenous (iv) route. Neutralizing antibody titers are expressed as log10 of the reciprocal value of the serum dilution giving protection to CPE. Averages and SEM of the titers obtained for each group are shown for each time point. Antibody titers measured by ELISA are expressed as log10 of the reciprocal value of the serum dilution giving an A405 of 40% of the positive control. Each point represents the mean titer and SEM of five animals.

Total BoHV-1-specific antibody responses in animals immunized with the BoHV-1ΔgEβgal inactivated vaccine did not differ significantly from those of bovines vaccinated with BoHV-1 LA virus (Figure 3b). Antibody titers were first detected at 14 dpv, with highest levels reached at 30 dpv (approximately 4.0) in both vaccinated groups.

Similarly, neutralizing antibodies were first detected in vaccinated bovines at 30 dpv, (titers of 2.04 and 1.8 for BoHV-1ΔgEβgal-immunized and BoHV-1 LA-immunized groups, respectively). The neutralizing antibody titers before challenge ranged between 1.2 and 1.1 (Figure 3a). No significant differences were found in the responses elicited by BoHV-1ΔgEβgal and BoHV-1 LA as inactivated antigens. Nasal swabs were analyzed for isotype profiles of the virus-specific antibody responses at the time of challenge. We found that 5/5 animals vaccinated with BoHV-1 and 4/5 animals vaccinated with BoHV-1ΔgEβgal exhibited anti-BoHV-1 IgG1 antibodies in nasal swabs (titer = 1.6), while no IgA antibodies were detected in the same samples (data not shown).

Assessment of the gE-specific antibody response could be effectively utilized to differentiate animals vaccinated with inactivated BoHV-1ΔgEβgal, inactivated BoHV-1 LA, as well as those infected with BoHV-1 LA virus. At 14 dpv, groups that were BoHV-1ΔgEβgal-vaccinated, mock-vaccinated, or sentinel animals were found to be negative for gE-specific antibodies. However, these animals developed anti-gE antibodies by 216 dpv, 30 days after challenge. Additionally, gE-specific antibodies were detected in animals vaccinated with inactivated BoHV-1 LA strain at 14 dpv and were also positive by the end of the experimental period (data not shown).

**Figure 4:**
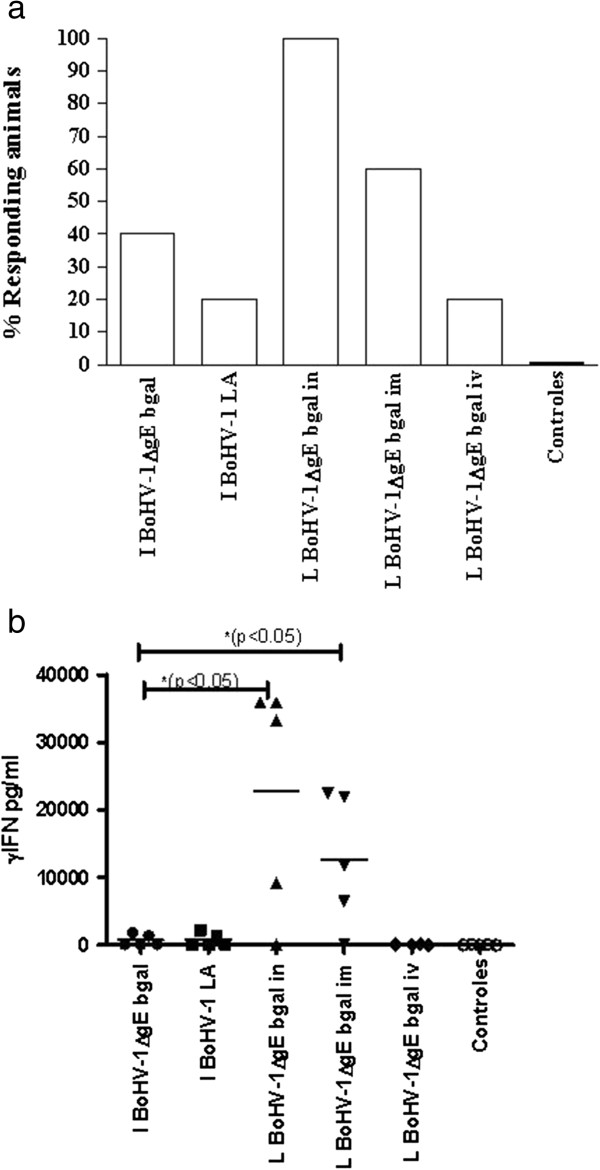
**Cellular response to BoHV-1 observed in vaccinated cattle. Cellular immune response at 7 dpv detected by (a) Lymphocyte proliferation test (LPT).** Values are expressed as the percentage of animals in each treatment group exhibiting significant (LPT response >3) lymphocyte proliferation after vaccination at 7 dpv. **(b)** IFNγ at 7dpv. Values are expressed as pg/ml. References in this figure are as described in Figure 3.

Cellular immune responses, detected by LPT and IFNγ production specific to BoHV-1 are shown in Figure 4. Animals produced a positive LPT (SI ≥ 3) in the BoHV-1ΔgEβgal group while a LPT response >3 was detected for one of the BoHV-1 LA-vaccinated animals. No significant differences were found in the responses elicited by BoHV-1ΔgEβgal versus BoHV-1 LA when administered as inactivated antigens, and none of the bovines belonging to the sentinel or mock-vaccinated groups showed positive reactions throughout the duration of the experiment (Figure 4a). Only low levels of IFNγ could be detected in the inactivated-vaccinated group during the evaluated period (Figure 4b).

Clinical monitoring of animals was performed daily, starting the day of vaccination until the end of the post-challenge observational period. No animals exhibited signs of BoHV-1 disease after vaccination. Bilateral nasal serous discharge was detected in the BoHV-1ΔgEβgal group at 2 days post-challenge (dpc) and for 11 days virus shedding was detected, with a maximum mean titer of 1.3 TCID_50_/ml at 8 dpc. In the BoHV-1 LA group, nasal discharge was mucous or serous. Virus shedding in mock-vaccinated animals peaked at 8 dpc (6.1 TCID_50_/ml) and serous to mucous nasal discharge lasted approximately 2 weeks. The protective responses were statistically different (p < 0.001) between vaccinated vs. mock-vaccinated animals, while no significant differences were found between animals vaccinated either BoHV-1ΔgEβgal or BoHV-1 LA.

### Assessment of efficacy BoHV-1ΔgEβgal virus as a live attenuated vaccine

Animals involved in this assessment were randomly allotted into five groups of five cattle each. Groups 1, 2 and 3 received 4 ml of 10^8.25^ TCID_50_/ml of BoHV-1ΔgEβgal virus by the IN, IM and IV routes, respectively. Group 4, the sentinel group, did not receive vaccine or challenge virus, but was housed with vaccinated animals beginning at 3 dpv, to evaluate horizontal transmission. Group 5 consisted of mock-vaccinated calves.

BoHV-1ΔgEβgal virus was shown to be attenuated. All animals vaccinated with BoHV-1ΔgEβgal, regardless of the inoculation route, exhibited minimal clinical symptoms of infection, with serous rhinitis as the only observed indicator of disease. No apathy, depression or loss of appetite was observed in these calves and their body temperatures never exceeded 40°C. Additionally, no detectable BoHV-1ΔgEβgal virus was found in the nasal or ocular secretions of the vaccinated calves. Only two animals belonging to the IN group shed virus, with viral titers of 1.5 TCID_50_/ml at 3 dpv, likely due to the existence of residual virus from the vaccination. No virus shedding was detected in the sentinel animals.

Analysis of the antibody response in animals vaccinated with BoHV-1ΔgEβgal indicated that detection of antibodies, measured by ELISA, started at 19 dpv in those vaccinated either IM or IV, while those vaccinated IN began exhibiting detectable antibody responses at 34 dpv, with significantly lower titers (p < 0.001) compared to the IM- and IV-vaccinated groups. On the day of challenge (42 dpv), the average antibody titers were 0.7 (±1.2) in the IN-vaccinated group and 2.36 (±1.2) and 2.05 (±0.3) in the IM- and IV-vaccinated groups respectively (Figure 3b). After challenge, antibody levels increase for all groups, reaching their maximal levels at 56 dpv with values of 3.7 (±0.3) for the IN-vaccinated group and 3.88 (±0.26) for the IM-vaccinated group (data not shown). While in the mock-vaccinated group animals displayed average antibody titers of 3.04 (±0.29) at 30 dpc, no antibodies were detected during the entire post-vaccination period in the sentinel group. Antibody responses were statistically different (p < 0.001) between IM/IV- vs. IN-vaccinated groups, and between vaccinated vs. mock-vaccinated animals, while no significant differences were found between animals vaccinated either IM or IV.

In agreement to the presence of total BoHV-1 antibodies, detection of virus neutralizing antibodies (VN) started at 19 dpv in animals vaccinated IM and IV. Titers found for IM and IV groups were significantly higher than in the IN-vaccinated group (p < 0.001). No VN titers were detected in the IN-vaccinated, mock-vaccinated or sentinel groups (Figure 3a).

At 14 dpv, all three groups of animals vaccinated with BoHV-1ΔgEβgal were found negative for presence of gE specific antibodies. After challenge with BoHV-1 LA strain, only animals from two vaccinated groups (IN and IM) turned to be positive for the presence of gE-specific antibodies.

The BoHV-1 specific T lymphocyte stimulation was evaluated using the LPT. At 7 dpv, the percentages of calves with a positive response to the LPT assay depended on the infection route. In the BoHV-1ΔgEβgal IN group, all calves produced a positive LPT response, while 60% and 20% of the IM- and IV-vaccinated groups were positive responders, respectively (Figure 4a). At 72 dpv (30 dpc), a positive response was seen in 80% of the calves in the IN-vaccinated group, and in 40% of the IM-vaccinated group (data not shown). The results of IFNγ production were in agreement with the data obtained with the LPT. When the production of IFNγ was evaluated at 7 dpv the calves that received the vaccine by IN spray demonstrated greater levels of IFNγ compared to those which received the IM vaccine (Figure 4b). The levels of IFN-γ were significantly higher (p < 0.05) in those animals that received the live virus vaccine by IM and IN routes, than those receiving the inactivated virus vaccine. Surprisingly, no detectable INF-γ response was found for the group vaccinated via IV.

**Figure 5:**
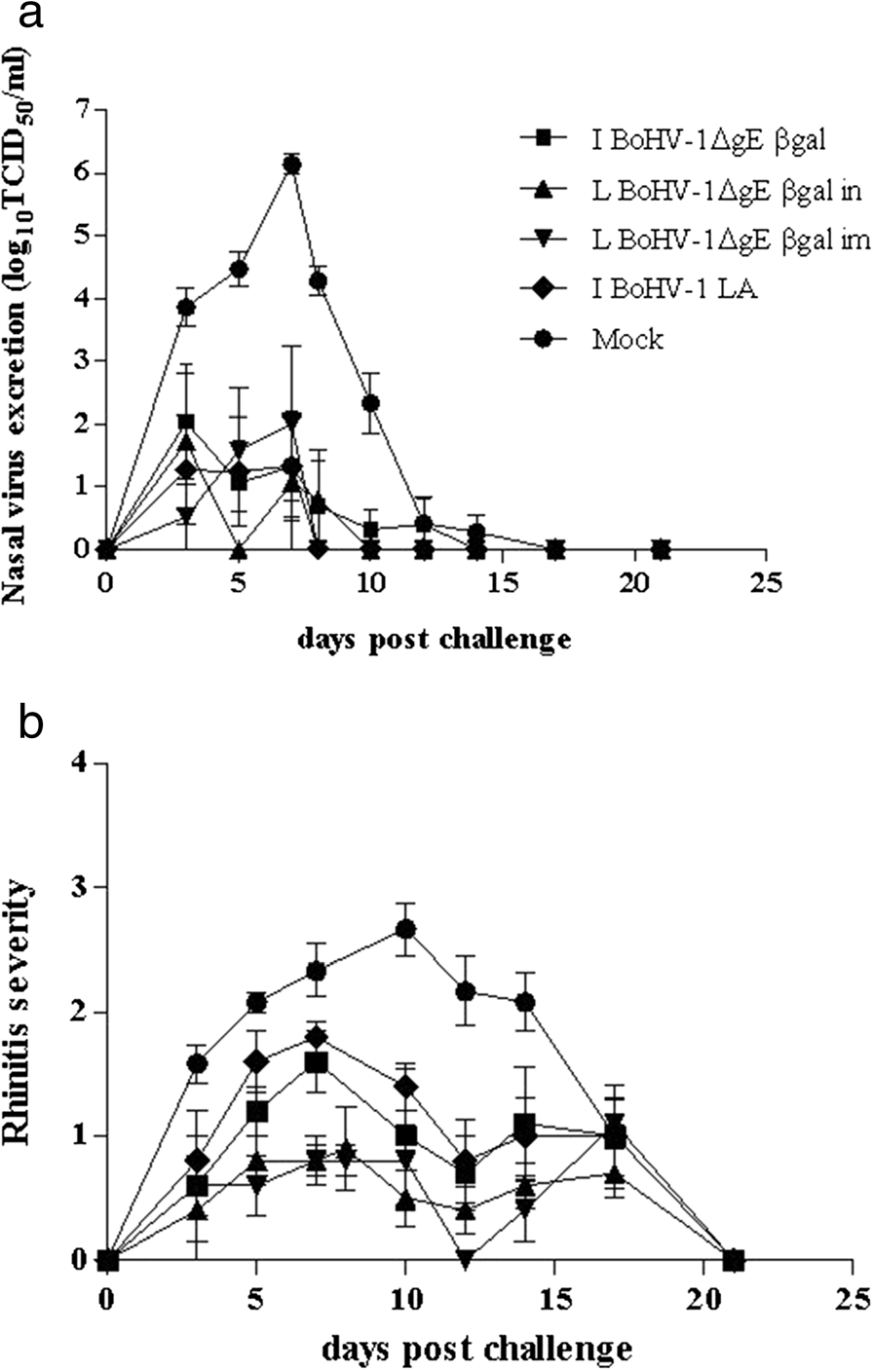
**Values of virus shedding (a) and rhinitis severity (b) in vaccinated cattle after challenge.** Virus shedding titers are expressed as log10 TCID50 per ml nasal fluid. **(b)** Each point in the graph is a average and SEM of the score taken in all the animals of each group. Rhinitis severity is score as follows: 0, absence; 1, slightly serous; 2, severely serous; 3, seromucous; 4, mucopurulent. References in this figure are as described in Figure 3.

Virus shedding titers during the post-challenge period of BoHV-1ΔgEβgal-vaccinated animals were significantly lower (p < 0.001) than in mock-vaccinated animals. Virus shedding of animals vaccinated via IN- and or IM showed maximum individual titers of approximately 10^5.5^ TCID_50_/ml. Mock-vaccinated calves shed virus for a period of 13 days and exhibited high individual titers (10^6.6^ TCID_50_/ml) (Figure 5a).

Clinical analysis of the mock-vaccinated animals demonstrated the presence of symptoms, characterized by bilateral serous rhinitis observed at 2 dpc that increased in severity, as manifested by severe seromucous rhinitis (score 2–4) by 8–11 dpc, returning to normal by 22 dpc. All vaccinated animals, regardless of the route of inoculation, displayed significantly less severe symptoms, suffering slight serous rhinitis (score 1 to 2) or absence of rhinitis (score 0) (p < 0.001) in comparison with the mock-vaccinated group (Figure 5b). No apathy or loss of appetite was registered in the vaccinated animals.

### Safety of BoHV-1ΔgEβgal LAV in pregnant animals

The pregnant cows that received the BoHV-1ΔgE-βgal virus by the IV route did not present abortions and all reached labor successfully. Animals only presented a transient and slight symptom manifestation of after the inoculation, without loss of appetite, depression or rise of body temperature. All the births reached term and none of the newborn calves showed detectable viral titers in their nasal secretions. Since their ninth day of life, we were able to detect in blood serum a titer of 1.6 of specific antibodies against BoHV-1 indicating the absorption of specific immunoglobulins from the maternal colostrum. BoHV-1ΔgE-βgal was shown to be completely innocuous not causing adverse collateral effects abortions/mummifications. Pregnant cows showed BoHV-1 specific antibody titers (≥ 1.6) until day 370 pv (data not shown). In addition, the LAV inoculated by IV route (group 3) induced long term immunity, since antibodies were detected during up to 370 days after a single immunization.

## Discussion

We describe here the development of a βgal-expressing gE-mutated BoHV-1 strain (BoHV-1ΔgEβgal). We characterized the use of BoHV-1ΔgEβgal as an immunogen in the formulation of both inactivated and LAV against BoHV-1. It is shown that BoHV-1ΔgEβgal, when used in either formulation, elicits an efficient immune response that protects animals against challenge with virulent wild-type BoHV-1. Importantly, antibody responses induced by BoHV-1ΔgEβgal virus (inactivated or live) can be serologically distinguished from those induced by wild-type virus, even at long term time-points after being exposed to infectious virus (30 dpc).

The viral gE ORF in BoHV-1ΔgEβgal was completely removed with the exception of the first 23 bp in its 5′ end and the last 67 bp in its 3′ end, and the β-gal gene was inserted in its place. Thus, BoHV-1ΔgEβgal differs from the mutant virus previously described by van Engelenburg *et al.*, which carries a deletion of the complete gE gene based on the Lam strain [9]; from the mutant described by Rebordosa *et al.*[10] and from the recombinant strain developed by Chowdhury *et al.* that contains 372 of the 575 aa of gE [32].

The βgal marker gene was chosen to be incorporated in the genome in order to facilitate the identification of the virus both for selection of recombinant clones and for the potential use in identification of the agent in samples derived from vaccinated animals. Removing the whole gE ORF constitutes a safety feature of the novel BoHV-1ΔgEβgal strain which also resulted in virulence attenuation. Due to the length of the insert, the addition of a βgal expressing cassette (4.5 kb) reduces to the minimum the probability of reversion to wild type forms of the BoHV-1.

Despite efforts made to generate a revertant, we were not able to obtain one from BoHV-1ΔgEβgal. Thus, the parental strain BoHV-1 LA was used as control for all vaccination experiments.

The vaccination schedule followed for testing efficacy for the inactivated vaccines was based on the official schedule of vaccination using inactivated vaccines against IBR disease (National Sanitary Service, SENASA). The official schedule mentioned involves first vaccination and a booster immunization after 21 days. It is known that 6 months after immunization with an inactivated BoHV-1 vaccine, virus-specific antibodies drop to a minimum value. In order to work with the maximum astringency, the calves were maintained and monitored during 6 months until challenge. On the other hand, manipulation of animals immunized using a LAV is considered a biohazard by the official authorities (National Advisory Commission of Agronomic Biotechnology, CONABIA). Thus, the experiments should be performed in closure and only after obtaining the corresponding authorization. For that, animals were maintained in a Type II biosafety facility. The experiment lasted the minimum period of time (42 days) enough for the vaccinated animals to develop a detectable immune response of a similar level to the one found in the field.

Inactivated BoHV-1ΔgEβgal displayed similar virus shedding period and titers as those recorded for the wild-type-based inactivated vaccine. These results are comparable to those communicated by Bosch *et al.*[21] and Kaashoek *et al.*[16] and ourselves [26]. A thorough evaluation of the specific BoHV-1 humoral and cellular immune responses demonstrates the antigenic integrity of the glycoprotein E-deleted bovine herpesvirus type 1 strain. Importantly, BoHV-1ΔgEβgal-vaccinated bovines were easily differentiated from BoHV-1 LA-vaccinated animals, mock-vaccinated and BoHV-1-challenged animals, indicating the efficacy of inactivated BoHV-1ΔgEβgal as a marker vaccine.

BoHV-1ΔgEβgal as a LAV was highly immunogenic, inducing a humoral immune response comparable to those induced by previous BoHV-1 attenuated vaccines [2,11,15,18,32,33].

Significantly, BoHV-1ΔgEβgal induced an antibody response that could easily be differentiated from that elicited by parental wild-type BoHV-1 LA virus. During the post-vaccination period of both inactivated and LAV BoHV-1ΔgEβgal studies, no anti-gE antibodies were detected until after challenge with the wild-type BoHV-1 virus, thus demonstrating its suitability as a DIVA marker vaccine.

The aim of the present work was to evaluate safety in terms of vertical or horizontal spreading of a novel BoHV-1 marker strain. Thus, an extremely high dose of BoHV-1ΔgEβgal was used for immunization. The minimum immunogenic and protective dose for its use in a commercial vaccine will be determined in future experiments. Live BoHV-1ΔgEβgal exhibits a noticeable attenuation, no virus was detected in nasal secretions during the post-vaccination period. This performance was better than other BoHV-1 recombinant strains containing gE deletions [2,32,33], which showed virus shedding from 10^4^ to 10^6^ TCID_50_/ml in a period from 6 to 8 days post-immunization. In order be considered safe, a LAV should not perpetuate in the cattle population [34]. Strube *et al.* reported that although the gE-deleted IBR vaccine strain Difivac® may be shed by immunized animals, it has a limited ability to pass from animal to animal [35]. In our study, although a high dose of virus was used to inoculate the animals (10^8.25^ TCID_50_/ml), no BoHV-1ΔgEβgal was recovered from the nasal secretions of vaccinated or sentinel animals although both groups cohabitated up to the 42 dpv. Furthermore, BoHV-1ΔgEβgal IV immunized pregnant cows did not transmit the recombinant virus to their newborns.

The IV route of inoculation is usually considered the most aggressive immunization or infection method during pregnancy, since it represents a direct access of any pathogen or drug to critical organs (ie, spleen, liver, lungs), and thus is the route of choice to test drug safety. Safety assays performed in pregnant cows demonstrated that vertical spreading of BoHV-1ΔgEβgal was null and newborn calves born to infected mothers showed detectable levels of anti-BoHV-1 maternal antibodies by day 9 after birth.

Two important features should be considered when comparing our results to those from Mars *et al.* their mutant virus did not carry a deletion of the complete gE ORF and cattle used in that study were 4 weeks-old calves [34]. Similar results were reported by van der Poel *et al.* using a BoHV-1ΔgE strain inoculated intramuscularly [36]. Once in the animals, BoHV-1ΔgEβgal showed absence of horizontal and vertical spreading most probably related to the low replication levels of the recombinant strain, although this should be clarified by future pathogenicity, latency and reactivation experiments.

Protection against challenge induced by the recombinant virus was evaluated in terms of reduction of clinical disease and, most importantly, reduction of shedding of challenge virus. Only half of the IM- or IN-vaccinated and challenged animals excreted virus, with titer values of 10^2.6^ to 10^5.5^ TCID_50_/ml during a 2 day period. The levels of excreted virus in a post-challenge period were similar to those reported by other authors [2,11,15,17,18,33,35]. Even though animals vaccinated with inactivated BoHV-1ΔgEβgal showed higher VN titers than those immunized with live attenuated virus (IM or IN routes), no differences were found on virus shedding following BoHV-1 viral challenge. Remarkably, despite the fact that no detectable VN titers were found in circulating blood, the animals vaccinated by intranasal route with BoHV-1ΔgEβgal virus were protected against challenge. This result may probably related to the local induction of virus-specific immunity, as mentioned for individuals immunized with the inactivated BoHV-1ΔgEβgal virus, but needs to be further confirmed.

As a whole, our results showed that the BoHV-1ΔgEβgal may be used both as an inactivated immunogen or as a LAV against BoHV-1 infection in cattle. This recombinant virus elicited an efficient immune response with both strategies, which can protect animals from challenge with virulent wild-type BoHV-1 and may be serologically differentiated from that induced by wild-type virus. Safety assays demonstrated that BoHV-1ΔgEβgal was not horizontally or vertically transmitted when administered as a LAV, even when very high doses were utilized. The minimum immunogenic and protective dose for its use in a commercial vaccine will be determined in future experiments.

## Conclusion

The BoHV-1ΔgEβgal strain is completely safe in terms of transmission to unvaccinated animals or environmental dissemination; BoHV-1ΔgEβgal is absolutely innocuous in pregnant cows and behaves as an attenuated BoHV1 strain in calves. Also, BoHV-1ΔgEβgal is protective against viral challenge and acts as an efficacious marker vaccine, since no antibodies were detected against gE protein during the post-vaccination period. Therefore, the new vaccine strain described here provides a useful combination of safety, attenuation, immunogenicity and DIVA capability.

## Competing interests

The authors declare that there are no competing interests.

## Authors’ contributions

SR, MP and AS designed the experiments, analyzed the data. SR, MP and MB drafted the manuscript together. SR and MP performed the experiments. PDM, VQ, PZ and JBV helped with *in vivo* experiments. MB, SC and CC participated in the interpretation of data and preparation of the manuscript draft. All authors read and approved the final manuscript.
